# Comorbidities in individuals with intellectual disabilities with and without hearing loss: a prospective cohort study

**DOI:** 10.1186/s12889-026-29321-w

**Published:** 2026-09-26

**Authors:** Awa Naghipour, Corinna Gietmann, Vincent Jankovic, Martin Scharpenberg, Susanna Marie Zielonkowski, Lukas Prein, Katharina Schwarze, Anja Neumann, Susanne Wasmuth, Karolin Schäfer, Christian Speckemeier, Sarah Schlierenkamp, Sandra Werner, Ross Parfitt, Anna Wiegand, Anna-Sophia Schwalen, Werner Brannath, Philipp Mathmann, Katrin Neumann

**Affiliations:** 1 Department of Phoniatrics and Paediatric Audiology, University Hospital Muenster, University of Muenster, Malmedyweg 13, Muenster, 48149 Germany; 2 Competence Center for Clinical Trials Bremen, University of Bremen, Bremen, Germany; 3 Institute for Health Care Management and Research, University of Duisburg-Essen, Essen, Germany; 4 Institute for Special Needs Education (d/Deaf and Hard of Hearing), University of Duisburg-Essen, Essen, Germany; 5 Essener Forschungsinstitut Für Medizinmanagement - EsFoMed GmbH, Essen, Germany; 6 Department of Otorhinolaryngology, Head and Neck Surgery, Johannes Wesling Klinikum Minden, Ruhr-University Bochum, Minden, Germany; 7 South African College of Music, University of Cape Town, Cape Town, South Africa; 8 Medical Faculty, Universitas Airlangga , Surabaya, Indonesia

**Keywords:** Intellectual disability, Hearing loss, Comorbidity, Sex, Age, ICD-11, Health equity, Multidisciplinary care

## Abstract

**Background:**

Both intellectual disability (ID) and hearing loss (HL) are associated with a high prevalence of certain comorbidities. However, a combined analysis and overview classified according to the International Classification of Diseases, 11th Revision (ICD-11), is lacking. HL is disproportionately common among individuals with ID and often remains undetected. We evaluated: (1) which comorbidities are most common in ID; (2) how disease prevalence differs between those with and without HL; (3) whether the degree of ID or HL influences the number of additional diseases, surgeries, or medication intake.

**Methods:**

This study is part of a prospective cohort study investigating effectiveness and feasibility of repeated universal hearing screenings and interventions for people with ID. We included 1,053 participants with ID (37% female, 63% male, and < 1% intersex; age range 1–90 years). Medical history assessment and on-site hearing testing were combined. Diseases were ICD-11-coded. Descriptive statistics and binary logistic regression models assessed associations between common diseases and age, sex, HL, and ID.

**Results:**

Nearly all comorbidities observed in participants with HL (prevalence > 5% [= 14]) were more frequent in participants with HL vs. without (13/14), eight showing odds ratios [OR] and 95% confidence interval [CI] > 1: visual impairment; hypertension; trisomy 21; obesity; mental, behavioural, or neurodevelopmental disorders; spinal conditions; diabetes; hypothyroidism. Logistic regression underscored associations between: (1) HL and visual impairment, trisomy 21, spinal conditions, hypothyroidism; (2) age and hypertension, obesity, diabetes; (3) female sex and trisomy 21, hypothyroidism. Epilepsy occurred similarly (OR 1.01, 95% CI: 0.65–1.56) and autism spectrum disorder (ASD) less often in the HL-subcohort (OR 0.42, 95% CI: 0.24–0.73). Trisomy 21 was 3–4 times more frequent in the HL-subcohort, showed a female preponderance and high rates of cardiovascular, metabolic, and visual diseases. Associated comorbidities (mean 2.75 vs 1.65) and multimorbidity (≥ two diseases, 63% vs. 42%) were higher in persons with HL than without, as was medication intake (mean 2.73 vs. 0.84).

**Conclusion:**

In our study individuals with ID and HL showed increased comorbidity, multimorbidity, and higher medication intake than those without HL. Heightened awareness and individualised, multidisciplinary management are essential in order to ensure equitable care and prevent secondary complications.

**Supplementary Information:**

The online version contains supplementary material available at https://doi.org/10.1186/s12889-026-29321-w.

## Introduction

Individuals with intellectual disabilities (ID) comprise approximately 1–2% of the global population, and a similar proportion of the German population, with a slightly higher prevalence reported for males (1.42%) than females (1.37%) [1–3]. Individuals with ID have repeatedly been reported to have a higher proportion of a variety of diseases [3–14], with earlier age of onset [5] and a higher mortality rate [4, 15–19] than in the general population. Alarmingly, Heslop et al. demonstrated that twice as many individuals with ID die of causes that could have been prevented by adequate health care than in the general population [15, 20]. Similarly, Thygesen et al. reported a standardised mortality ratio (SMR) of 2.62 (95% CI, 2.51–2.73) for preventable deaths and 6.00 (5.72–6.29) for treatable causes of death [19]. Individuals with ID also carry a greater risk of unidentified morbidities [21]. Baxter et al. found that 51% of study participants with ID who underwent health checks had newly discovered health needs, 63% of them one, 25% two, and 12% more than two [21].

Hearing loss (HL) poses a specific health challenge for individuals with ID [22]. The prevalence of HL is reported to be higher than in the general population, and systematic gaps in both diagnostics and further care have been found [22, 23].

HL affects approximately 20% of the world's population and 34.5% of the German population, with prevalence increasing with age and further exacerbated by demographic change [22, 24]. The significance of HL is underscored by the fact that, according to the Global Burden of Disease Study 2019, it is the third most common reason for years lived with disability and was ranked first among sensory disorders [24]. In another arm of our study, we found a negative association between severity of HL and hearing-related quality of life [25].

Schrojenstein Lantman-De Valk et al. already found a significantly higher prevalence of sensory impairments in individuals with ID in the year 2000 [12]. They suggested that the limited communication abilities of individuals with ID are further compromised when sensory impairments remain undetected and untreated.

One of the first population-based studies to examine the prevalence of HL in individuals with ID was carried out by Meuwese-Jongejeugd et al. [23]. Their results suggested a significantly higher prevalence of HL in individuals with ID (30.3%) than in the general population (16–17%), with 47.6% of cases undiagnosed prior to the study. The authors further identified Down syndrome and age as pertinent risk factors for HL [23]. The relevance of HL has also been demonstrated in the context of the Special Olympic games, an athletic competition for people with intellectual disabilities [26–28]. An overview of hearing screening measurements performed at the special Olympics in 2007–2017, published in 2022, revealed a high prevalence of HL [28]. Over 100,000 hearing screening measurements were conducted, finding “cerumen impaction [in 40.7%], middle ear problems in 29.5% of those who failed hearing screening, and hearing loss confirmation in 26.9%” [28]. While cerumen impaction was frequent among those who failed the screening (40.7%), such obstructions typically result in only a mild, often reversible HL (approx. 16 dB) [29]. Twenty-three point five percent of participants at the 2006 German Special Olympic games failed hearing screening tests, for which a sensitivity of 100% (95% CI, 98.1% to 100%) and a specificity of 98% (95% CI, 95.1% to 100%) was confirmed [26]. As many as 38% failed hearing screening overall in the 2004 German Special Olympic games [27].

The above-mentioned data underscore the relevance of (1) multiplied disease occurrence in individuals with ID and (2) HL in persons with ID. Without enough sufficiently-specific data, marginalised populations, such as individuals with ID, will continue to be under-represented and subject to insufficient medical care. This study aimed to address the gaps in existing data and care concerning individuals with ID and HL by answering the following questions within our cohort: (1) which comorbidities are most frequently observed in persons with ID and HL; (2) which comorbidities occur more often in this group than in individuals with ID but no HL; (3) whether the severity of both ID and HL influence the quantity of diseases, surgeries, or medication intake. In answering these questions, special consideration is given to factors such as sex and age. Investigating comorbidities in individuals with ID and HL at the intersections of sex and age adds nuance and novelty to the currently limited evidence base.

## Methods

### Study design and setting

This prospective, age-stratified cohort study, partially population-based in design, was conducted in the geographical area of North Rhine-Westphalia.

### Participants

A total of 1,053 participants with ID (mean age 24 years, age range 1–90 years) were included in the outreach group, of whom 390 were female (37%), 662 male (63%), and 1 intersex (< 1%). Additionally, 141 individuals with ID were invited for assessment within clinics as part of a control cohort. However, none of the 12 individuals from the clinical control group who accepted the invitation for hearing screening provided written consent for participation. Therefore, the absence of a control group limits comparative analyses between clinical and outreach settings, and only data from the outreach group is presented in this analysis. The project took place between January 2020 and July 2024, during which time recruitment, screening, data collection, and one-year follow-up took place. The inclusion criteria required that participants had a diagnosed ID and were insured with the project partner, the insurance company AOK-Rhineland. Ethical approval was granted by the ethics committee of the Medical Association of Westphalia-Lippe and the University of Muenster (2020–843-f-S), and complied with the Declaration of Helsinki [30]. The study was registered with the German Clinical Trials Register (DRKS-ID: DRKS00024804). A written project description in plain language was provided to participants with ID in order to ensure that they thoroughly understood and took part in the study voluntarily. The consent form was signed by the participants themselves and/or their legal guardian, depending upon participants’ age, decision-making capacity, and status of guardianship. Caregivers were also released from confidentiality in order to legitimately provide further necessary information relevant to the study. The ability of participants to terminate the study at any time was emphasised, and the work was carried out only on the basis of the ongoing consent of participants and legal guardians throughout.

Participant information was gathered using a questionnaire that had been developed by the study group and was administered to caregivers and, where applicable, to participants typically before the first screening measurements took place. Hearing assessments were performed in the living, educational, and working environments of the participants, creating a setting that was more familiar than a sterile clinical environment. To confirm that the measurement environment was as quiet as possible, the background noise level was measured using a sound level meter to ensure that a noise level of 40 dB(A) was not exceeded. In cases where the sound level meter recorded noise levels of 40 dB(A) or higher, the hearing screening measurements were interrupted and/or relocated to a less noisy environment [31].

The assessments entailed three steps: screening, further diagnostic measurements where necessary, and treatment in cases where HL was identified. The audiometric assessments were performed using the handheld audiometry device Sentiero® (PATH MEDICAL GmbH). The screening process included otoscopy, tympanometry, pure-tone audiometry (air and bone conduction; 0.5–8 kHz), and transiently evoked otoacoustic emissions (TEOAE). For participants unable to undergo conventional audiometry, image-based methods (Multiple-choice Auditory Graphic Interactive Check, MAGIC) or distorted product otoacoustic emissions (DPOAE) were utilised. Inconclusive results were further investigated using brainstem evoked response audiometry (ABR), auditory steady state response (ASSR), DPOAE growth functions, or speech audiometry (Freiburger monosyllabic test or the Mainzer Audiometric Test for Children, MATCH). Following the WHO criteria [32], a hearing impairment was defined as a mean hearing threshold of ≥ 20 dB HL across the frequencies 0.5, 1, 2, and 4 kHz, at both T0 and T1. While isolated hearing losses in the high, mid, or low-frequency ranges were documented, only those meeting the WHO criteria were classified as manifest HL for the purpose of determining prevalence [31, 33]. A detailed description of the entire methodological procedure is published in the study protocol by Schwarze et al. (2021) [34].

### Questionnaire

The questionnaire developed by the study group comprised two parts: medical history and assessment of hearing-related quality of life. The part of the study reported here drew upon data from the following subcategories of the first section: general health status, severity of ID as evaluated by the caregivers, ear-specific history, general surgeries, medication, and relevant pre-existing diseases.

The questionnaire section on medical conditions covered a variety of diseases: cardiovascular, neurological, psychiatric, eye, ear, spinal, musculoskeletal, soft tissue, lipo-metabolic; malformations of muscles, skeleton, ears, head, or neck; syndromes associated with HL, such as Alport, Waardenburg, Alström, Usher, Pendred, Cogan-I, and Refsum syndromes; familial HL; and open sections to specify other relevant diseases.

The diseases reported were organised and coded according to the most recent International Classification of Diseases, 11th Revision (ICD-11), by the WHO. The complete list of distinct disease-groups and diseases amounted to 21 overall ICD-11 categories and 290 subcategories.

### Manifest HL

Because the program of hearing screening, diagnostics, and intervention was repeated after one year, we were able to distinguish between transient and permanent HL, as well as progressive HL evolving between the first (T0) and second (T1) screening timepoints. Data between T0 and T1 were collected via telephone interviews conducted at least 6 months after T0 to determine the frequency and results of external diagnostic and therapeutic procedures, including hearing aid fitting by our program-affiliated hearing specialists. The reasons for non-utilization of diagnostic or therapeutic measures were recorded using pre-defined categories (e.g., refusal by caregivers/participants, lack of appointments, or missing referral letters) [33, 35]. Manifest HL was defined as permanent (present at T0 and T1) or progressive HL (present at T0, and worsened at T1, or not present at T0 and sensorineural, mixed, or unspecified HL at T1) identified across those two subsequent assessments (algorithm further outlined in supplementary file: Algorithm – Manifest Hearing Loss). Transient or reversible HL was defined as present at either T0 or T1 and was excluded from the presented analysis on manifest HL.

### Statistical methods

Relevant items of the pseudonymised questionnaire (diseases, surgeries, medication, previous knowledge of HL) and hearing status were organised and analysed statistically using descriptive statistics (Standard Deviation [SD], mean, Spearman’s Rho) in R (Version 4.4.1, 2024–06–14 ucrt). We describe diseases present in 3% or more of the overall cohort and 5% or more in the subcohorts (with and without HL) in order to achieve sufficient sample size. Associations between HL and individual diseases were evaluated using odds ratios (ORs) with 95% confidence intervals (CIs). Comparisons between groups were conducted using a chi-squared test or Welch’s t-test. All of the tests are two-sided, and a formal significance level of α = 0.05 was used. Binary logistic regressions were performed on data relating to the most common diseases overall (disease prevalence > 3% in the cohort [= 15]) in order to assess the association between each disease and age, sex, HL, and grade of ID [7]. Participants with missing data for a specific covariate (e.g., undetermined grade of ID or unclear hearing status) were excluded from the corresponding analysis. The analyses regarding comorbidities in individuals with ID and HL presented in this manuscript were not the primary endpoint of the overall HörGeist-project for which an initial power calculation and correction for multiple testing was performed. We therefore consider the analyses presented here to be secondary and exploratory. Consequently, none of the reported p-values or confidence intervals were adjusted for multiple testing.

## Results

### Demographics

Table 1 summarises participants’ demographics, caregiver-rated ID, and hearing status assessed during the study.

**Table 1:** Overview of the cohort stratified by sex, age, with/without manifest HL, degree of ID (as evaluated by caregivers), and degree of manifest HL

**Variables**	With HL *n* = 417	Without HL *n* = 554	Unclear *n* = 82	Overall *n* = 1,053
Sex				
Male	248 (59%/*37%)*	353 (64%/*53%*)	61 (74%/*9.2%)*	662 (63%/*100%*)
Female	169 (41%/*43%*)	200 (36%/*51%*)	21 (26%/*5.4%)*	390 (37%/*100%*)
Intersex	0 (0%/*0%*)	1 (0.2%/*100%*)	0 (0%/*0%*)	1 (< 0.1%/*100%*)
Age*				
0–5	52 (12%/*23%*)	147 (27%/*64%*)	32 (39%/*14%*)	231 (22%/*100%*)
6–11	64 (15%/*26%*)	159 (29%/*64%*)	26 (32%/*10%*)	249 (24%/*100%*)
12–17	38 (9.1%/*24%)*	110 (20%/*71%*)	8 (9.8%/*5.1%*)	156 (15%/*100%*)
18–34	28 (6.7%/*26%)*	73 (13%/*68%*)	7 (8.5%/*6.5%*)	108 (10%/*100%*)
35–49	57 (14%/*62%*)	30 (5.4%/*33%*)	5 (6.1%/*5.4%*)	92 (8.7%/*100%*)
50–69	144 (35%/*80%*)	34 (6.1%/*19%*)	3 (3.7%/*1.7%*)	181 (17%/*100%*)
≥70	34 (8.2%/*94%*)	1 (0.2%/*2.8%*)	1 (1.2%/*2.8%*)	36 (3.4%/*100%*)
Degree of ID				
Mild	75 (18%/*27%*)	178 (32%/*65%*)	20 (24%/*7.3%*)	273 (26%/*100%*)
Moderate	184 (44%/*44%*)	209 (38%/*49%*)	35 (43%/*8.2%*)	428 (41%/*100%*)
Severe	129 (31%/*45%*)	135 (24%/*47%*)	23 (28%/*8.0%*)	287 (27%/*100%*)
(missing**)	29 (7.0%/*45%*)	32 (5.8%/*49%*)	4 (4.9%/*6.2%*)	65 (6.2%/*100%*)
Degree of HL				
Mild	156 (37%)	-	-	156 (15%)
Moderate	87 (21%)	-	-	87 (8.3%)
Moderately severe	54 (13%)	-	-	54 (5.1%)
Severe	22 (5.3%)	-	-	22 (2.1%)
Profound	5 (1.2%)	-	-	5 (0.5%)
Complete	9 (2.2%)	-	-	9 (0.9%)
(missing**)	25 (6.0%/*23%*)	-	82 (100%/*77%*)	107 (10%/*100%*)
Unilateral HL	59 (14%/*9.6%)*	-	-	59 (5.6%/*100%*)

Percentages are reported first as column percentages (within the “with HL”/“without HL” subgroups) and secondly (in italics) relative to the entire cohort (row percentages). *Narrower age intervals stated in order to be able to distinguish life-cycle phases, such as puberty and menopause  **Participants with missing data for a specific covariate (e.g., undetermined grade of ID or unclear hearing status) were excluded from the corresponding analysis

### Manifest HL

Our findings represent associations observed within our cohort and should be considered hypothesis-generating. Therefore, the results do not imply causality. Among the 1,053 participants, 443 (42%) had no HL, 417 (40%) a manifest HL, 111 (11%) a transient HL, and in 82 (8%) the hearing status remained unclear. For the subsequent analysis, participants with transient HL were categorised as having no manifest HL. Among participants with manifest HL, the type of HL was conductive in 22% of cases, sensorineural in 42%, mixed in 12%, and remained unspecified in 25%. Our data exhibited an increase in HL prevalence, as expected, with age (Table 1) from 23% (0–5 yrs) to 94% (≥ 70 yrs). Of the 417 individuals with manifest HL, approximately 1/3 had mild HL (*n =* 156, 37%), 21% moderate (*n =* 87), 13% moderately-severe HL (*n =* 54), 5.3% (*n =* 22) severe, 1.2% (*n =* 5) profound, and 2.2% (*n =* 9) complete HL**.** For 25 participants (6%), HL was identified, but the exact degree of severity could not be determined due to participant restlessness or insufficient data points. Fourteen percent (*n =* 59) showed unilateral HL. Caregivers evaluated the degree of ID as mild in 18% (*n =* 75), moderate in 44% (*n =* 184), and severe in 31% (*n =* 129), with 7% (*n =* 29) missing due to incomplete records. Among participants with manifest HL, only 2.4% had persistent non-removable cerumen obturans at both the T0 and T1 screening assessments. The algorithm defining manifest HL excluded those with transient HL, suggesting that transient conditions were unlikely to have substantially influenced the prevalence of manifest HL.

### The ten most common ICD-11 disease categories

The ten most common ICD-11 disease categories (prevalence > 5% in the overall cohort) reported in the overall study cohort (with ID, with and without HL) were: diseases of the endocrine, nutritional, or metabolic system (*n =* 268, 25.5%); developmental anomalies (*n =* 263, 25%); nervous system disorders (*n =* 244, 23.2%); mental, behavioural, or neurodevelopmental disorders (*n =* 243, 23.1%); visual system disorders (*n =* 187, 17.8%); circulatory system disorders (*n =* 172, 16.3%); musculoskeletal system or connective tissue disorders (*n =* 99, 9.4%); symptoms, signs, or clinical findings not elsewhere classified (*n =* 84, 8%) (e.g., auditory hallucinations, tinnitus); ear or mastoid process disorders (*n =* 57, 5.4%); and factors influencing health status or contact with health services (*n =* 57, 5.4%) (e.g., ear or visual conditions documented in the medical history).

**Fig. 1:**
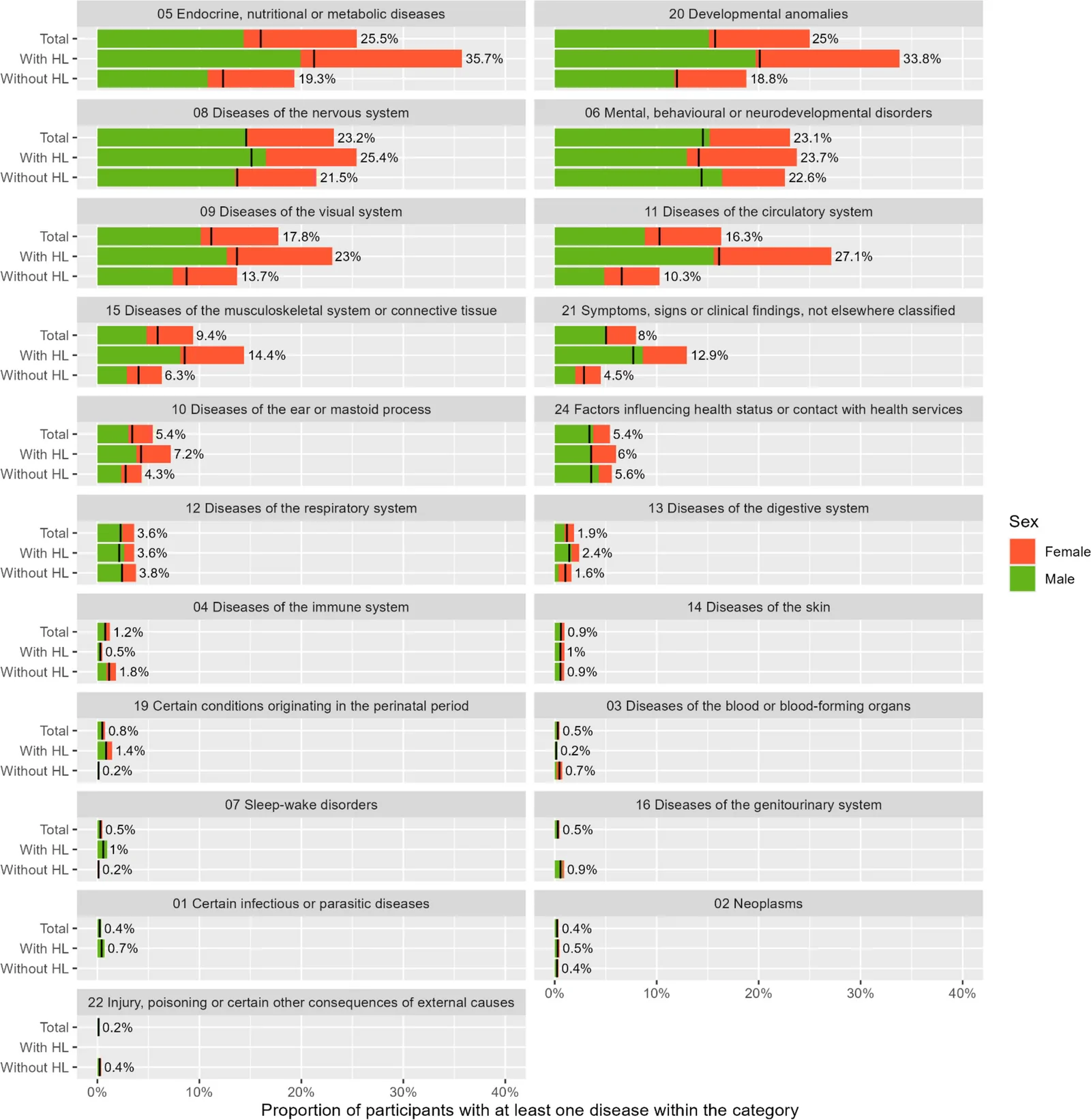
All 21 ICD-11 disease categories in the overall population, contrasting HL and sex differences. Percentual sex differences are highlighted within each disease category. The black vertical line marks the position, in which sex distribution among individuals with the disease would match that of the overall cohort or the respective subcohort (with/without HL), representing the expected ratio if the diseases were independent of sex. If the black vertical line falls within either the orange or green area, it indicates a higher disease prevalence in that sex.

The most common disease categories (ICD-11) identified among participants with and without HL showed the same categories but differed in prevalence. The comparison of all ICD-11 disease categories detected in the overall study cohort, contrasting those with and without HL, is illustrated in Fig. 1, highlighting sex differences within each disease category.

### Most common disease subcategories

The most common subcategories identified in the overall cohort (disease prevalence > 3% in the cohort, 15 subcategories) were: visual system diseases; epilepsy; obesity; ASD (autism spectrum disorder); hypertension; trisomy 21; neuromuscular disorders; nutritional disorders; skeletal anomalies; spinal conditions; mental, behavioural, or neurodevelopmental disorders; myocardial diseases; family history of eye/ear conditions; diabetes; and hypothyroidism. In the cohort without HL (disease prevalence > 5% in the subcohort, 9 subcategories), the most common subcategories were ASD; visual system diseases; epilepsy; obesity; nutritional disorders; neuromuscular disorders; skeletal anomalies; family history of eye/ear disorders; and myocardial diseases.

In the cohort with HL (disease prevalence > 5% in the subcohort, 14 subcategories), the most common subcategories were visual system diseases; hypertension; obesity; trisomy 21; epilepsy; neuromuscular disorders; skeletal anomalies; mental, behavioural, or neurodevelopmental disorders; spinal conditions; nutritional disorders; diabetes; myocardial diseases; hypothyroidism; and family history of eye/ear disorders.

### Comparison of common disease subcategories in participants with and without HL

**Fig. 2:**
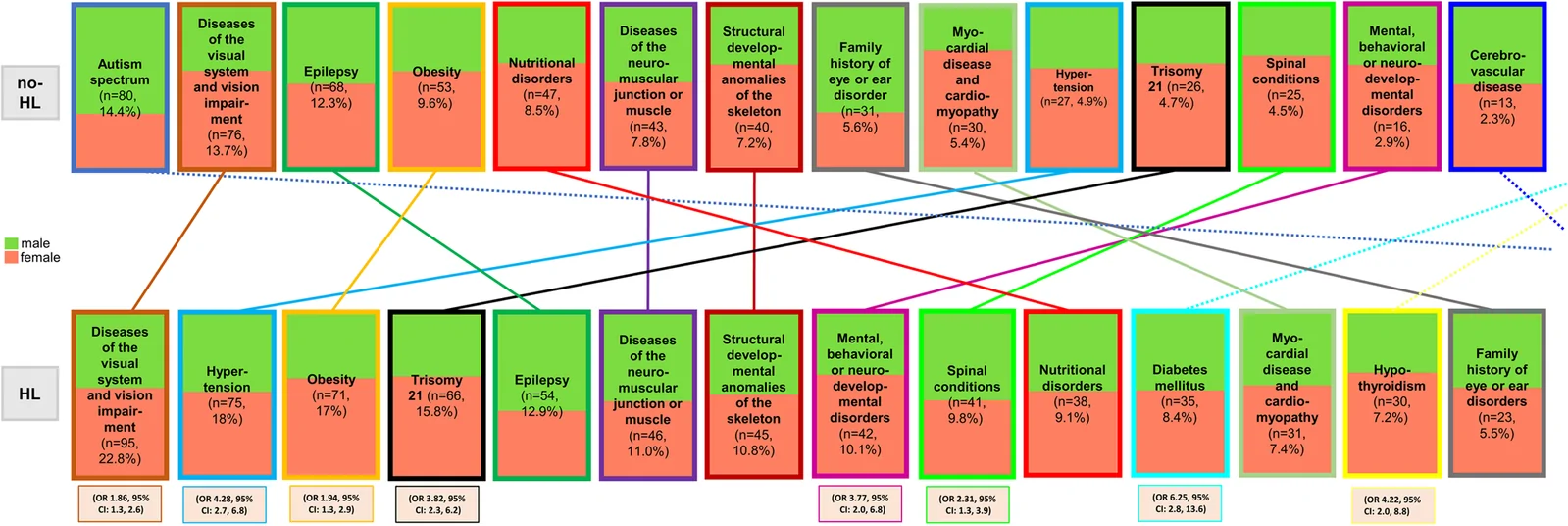
Comparison of top 14 most common disease subcategories in participants with and without HL. Upper row: order in participants without HL. Lower row: order in participants with HL. Color frames indicate each disease’s position per group. Straight lines show position changes, dotted lines indicate diseases exiting the top 14 list. Background boxes show sex differences: orange = female, green*=* male. Eight conditions differing between the two groups (OR and 95% CI > 1) are highlighted in the HL row.

Nearly all comorbidities found in participants with HL occurred more frequently in those with than without HL (13 out of 14), eight of which showed an OR and 95% CI > 1 (Fig. 2).

Visual impairment was found in 22.8% of participants with ID and HL vs. 13.7% without HL (OR 1.86, 95% CI: 1.3, 2.6), suggesting a one- to twofold higher occurrence in participants with HL. Hypertension occurred in 18% with HL vs. 4.9% without (OR 4.28, 95% CI: 2.7, 6.8), suggesting a fourfold increase. Trisomy 21 was detected in 15.8% with HL and 4.7% without (OR 3.82, 95% CI: 2.3, 6.2), thus occurring three to four times more often.

Obesity, among the four most common diseases in both groups, was observed nearly twice as often in participants with HL (17%) as in those without (9.6%) (OR 1.94, 95% CI: 1.3, 2.9). Mental, behavioural, or neurodevelopmental disorders were observed in 10.1% with HL vs. 2.9% without (OR 3.77, 95% CI: 2.0, 6.8); spinal conditions in 9.8% with HL vs. 4.5% without (OR 2.31, 95% CI: 1.3, 3.9); diabetes in 8.4% with HL vs. 1.4% without (OR 6.25, 95% CI: 2.8, 13.6); and hypothyroidism in 7.2% vs. 1.8% without (OR 4.22, 95% CI: 2.0, 8.8). All of these diseases except spinal conditions were more commonly observed in female than in male participants within the HL subgroup (Fig. 2), especially relevant in regard to trisomy 21 (*p* = 0.034, Chi-squared test).

Similar proportions of participants with HL (12.9%) and without HL (12.3%) reported epilepsy, underscoring its importance in both groups (OR 1.06, 95% CI: 0.7, 1.6). ASD was found less often in participants with HL than without (OR 0.28, 95% CI: 0.1, 0.5).

### Trisomy 21

Trisomy 21 is a known risk factor for HL [23]. We therefore analysed co-occurring diseases and HL in this group separately. Among 98 participants with trisomy 21, 51% were male, 49% female, mean age was 25 yrs (SD 22). The most common comorbidities (> 5% of the trisomy 21 subgroup) were diseases of the visual system and visual impairment (*n =* 23, 23.5%); obesity (*n =* 21, 21.4%); hypothyroidism; myocardial diseases (each *n =* 12, 12.2%); nutritional disorders (*n =* 8, 8.2%); neuromuscular disorders (*n =* 7, 7.1%); hypertension (*n =* 7, 7.1%); middle ear or mastoid disorders (*n =* 6, 6.1%); skeletal anomalies (*n =* 6, 6.1%); dyslipidaemias (*n =* 5, 5.1%); and spinal conditions (*n =* 5, 5.1%).

Sixty-seven percent (*n =* 66) of participants with trisomy 21 within our study were diagnosed with manifest HL but only 27% (*n =* 26) of those without. The most common type of HL found within this group was conductive (24%), followed by sensorineural (18%), and mixed HL (10%). In 18%, the type of HL remained unspecified.

## The extent of medication intake, (multi-)morbidity, and surgery

### Medication intake in participants with and without HL

The mean number of medications taken by participants with HL was 2.73 (SD 3.04) vs. 0.84 (SD 1.56) in those without HL, and thus higher in the subgroup with HL (*p <* 0.001, Welch test). The mean number of surgeries did not differ substantially*.* A small association was observed between the grade of HL and medication intake overall (Spearman’s Rho (95%-CI) 0.162 (0.06, 0.262)) and especially in females (Spearman’s Rho (95%-CI) 0.207 (0.044, 0.359)).

### (Multi-)morbidity in participants with and without HL

**Fig. 3:**
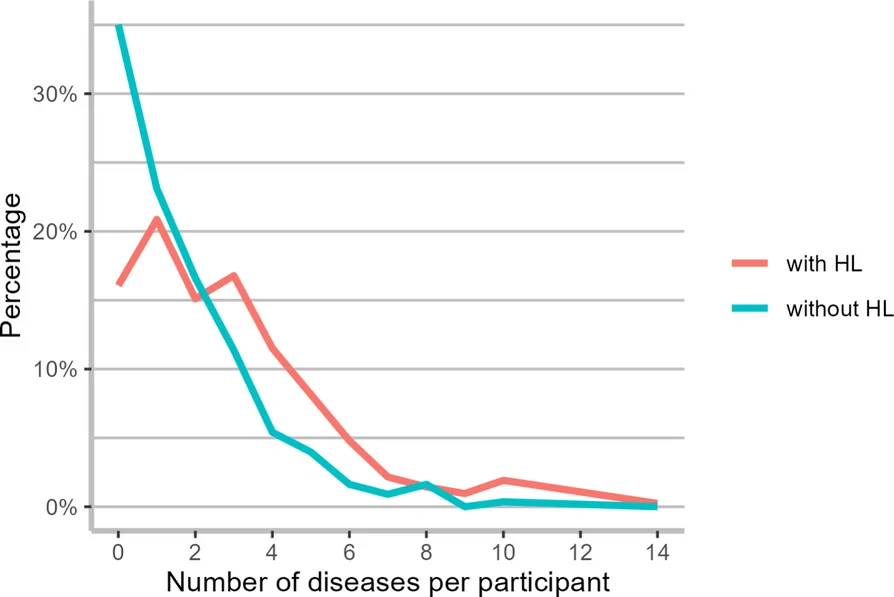
Number of diseases in participants with and without HL

The mean number of additional diseases was 2.75 (SD 2.35) in participants with HL vs. 1.65 (SD 1.87) without, and therefore higher in the subgroup with HL (*p <* 0.001, Welch test). Multimorbidity (two or more chronic diseases [36]) was observed in 49.8% (*n =* 524) of the cohort, 21 percentage points higher in those with ID and HL (63%) than in those without (42%) (*p <* 0.001, Chi-squared test). The highest number of diseases in a single participant was 14 (Fig. 3). Among those with ID and HL, 16% had no additional diseases, 21% had one, and 63% had two or more, vs. 35% (none), 23% (one), and 42% (two or more) without HL. A small association was observed between the number of comorbidities and the grade of HL (Spearman’s Rho (95%-CI) 0.192 (−0.036, 0.34)), as well as the number of comorbidities and degree of ID (Spearman’s Rho (95%-CI) 0.175 (0.019,0.322)) in females.

### Previously undetected manifest HL

Information regarding previous knowledge of HL was available in 1,003 participants. Eighty-five percent (*n =* 856) of these participants stated that HL was not previously known. However, 40% (*n =* 400) were diagnosed with a manifest HL, and 29% (*n =* 290) were found to have manifest, previously overlooked HL. Therefore, of all participants with manifest HL identified in this study, 72.5% had not been previously diagnosed. This group had a slight female preponderance (*p* = 0.41, Chi-squared test): 26% of male participants with previously undetected HL vs. 30% female participants.

### Comorbidities in context: associations with sex, HL, degree of ID, and age

A logistic regression analysis was performed in order to contextualise the overall most common disease subcategories (disease prevalence > 3% in overall study cohort, [15 subcategories]) portraying each disease subcategory’s association with sex, HL, degree of ID, and age group, while controlling for the other variables (Table 2).

**Table 2:** Logistic regression of disease subcategories and their association with sex, HL, degree of ID, and age

	**Sex**	Degree of ID*			**HL**		**Age group**	
**Disease**	Ref: Male	Mild	Moderate	Severe	Ref: without	0–17	18–49	50 +
Diseases of the visual system and visual impairment ᵃ	1.36 (0.95,1.93)	Ref	1.16 (0.75,1.81)	1.23 (0.76,2.01)	**1.58 (1.06,2.34)**	Ref	1.32 (0.83,2.06)	**1.6 (1,2.54)**
Epilepsy or seizures, unspecified ᵇ	0.97 (0.64,1.45)	Ref	1.54 (0.89,2.78)	**3.36 (1.94,6.03)**	1.01 (0.65,1.56)	Ref	1.04 (0.61,1.71)	0.97 (0.54,1.69)
Obesity ᶜ	1.49 (0.99,2.24)	Ref	0.89 (0.54,1.48)	1.23 (0.71,2.15)	0.95 (0.59,1.51)	Ref	**3.83 (2.25,6.54)**	**6.11 (3.52,10.78)**
Autism spectrum disorder (ASD) ᵈ	***0.5 (0.29,0.82)***	Ref	1.85 (1.03,3.46)	**2.55 (1.4,4.8)**	***0.42 (0.24,0.73)***	Ref	***0.23 (0.09,0.47)***	***0.09 (0.01,0.31)***
Hypertension ᵉ	1.01 (0.61,1.65)	Ref	1 (0.56,1.8)	0.85 (0.41,1.75)	1.02 (0.56,1.87)	Ref	**6.55 (2.69,17.54)**	**37.84 (16.72,98.43)**
Complete trisomy 21 ᶠ	**2.18 (1.37,3.5)**	Ref	1.82 (0.96,3.67)	1.82 (0.91,3.79)	**4.73 (2.78,8.25)**	Ref	0.92 (0.5,1.65)	***0.49 (0.25,0.9)***
Diseases of neuromuscular junction or muscle ᵍ	1.07 (0.66,1.72)	Ref	2 (0.98,4.4)	**4.18 (2.1,9.1)**	**1.67 (1.01,2.77)**	Ref	***0.34 (0.15,0.7)***	0.56 (0.28,1.06)
Nutritional disorders ʰ	**1.6 (1,2.55)**	Ref	1.49 (0.81,2.83)	1.7 (0.89,3.33)	1.13 (0.66,1.89)	Ref	0.66 (0.33,1.23)	0.58 (0.28,1.14)
Structural developmental anomalies of the skeleton ⁱ	1.33 (0.83,2.11)	Ref	1.18 (0.65,2.23)	**1.87 (1.01,3.59)**	1.36 (0.81,2.27)	Ref	1.12 (0.6,2.02)	1.19 (0.63,2.21)
Conditions associated with the spine ʲ	1.22 (0.71,2.07)	Ref	1.68 (0.8,3.88)	**3.02 (1.41,7.07)**	**1.83 (1.02,3.34)**	Ref	1.93 (0.98,3.7)	1.79 (0.89,3.6)
Mental, behavioural or neurodevelopmental disorders ᵏ	1.62 (0.91,2.91)	Ref	1.09 (0.53,2.34)	1.47 (0.65,3.41)	1.6 (0.81,3.3)	Ref	**5.49 (2.3,14.05)**	**9.3 (4,23.92)**
Diseases of the myocardium or cardiac chambers and cardiomyopathyˡ	**1.91 (1.12,3.3)**	Ref	1.52 (0.73,3.41)	**2.78 (1.31,6.29)**	1.03 (0.56,1.91)	Ref	0.73 (0.31,1.58)	1.7 (0.84,3.43)
Family history of eye or ear disorders ᵐ	0.66 (0.34,1.23)	Ref	0.95 (0.47,1.98)	0.89 (0.41,1.93)	1.46 (0.76,2.76)	Ref	***0.26 (0.08,0.66)***	***0.33 (0.12,0.81)***
Diabetes ⁿ	1.26 (0.64,2.46)	Ref	0.48 (0.23,1.02)	0.45 (0.16,1.16)	2.21 (0.94,5.69)	Ref	**6.18 (1.67,29.24)**	**21.99 (7.02,98)**
Hypothyroidism ᵒ	**2.3 (1.2,4.53)**	Ref	1.12 (0.51,2.67)	1.04 (0.4,2.75)	**2.95 (1.35,6.86)**	Ref	**3.16 (1.33,7.73)**	**2.63 (1.1,6.6)**

Logistic regression of the most common disease subcategories for the whole sample (disease prevalence > 3% of the study cohort, [= 15]) in individuals with ID and HL and their association with sex, HL, degree of ID, and age group. Odds ratios are indicated in parentheses. OR and 95% CI > 1 is highlighted in bold. Reversed associations are highlighted in *bold and italic.* ICD-11 codes corresponding to each subcategory are listed below the table, as indicated by the superscript letters (a–o)

^*^As evaluated by caregivers; ICD-11 codes for each disease subcategory: ᵃ9E1Z, 9D9Z, 9D00.0, 9C8Z, 9B10, 9D90, 9D00.1, 9D00.2, 9C6Z, 9C84.Z, 9B11.0, 9B71.0Z, 9C40.BZ, 9C82.4; ᵇ8A6Z; ᶜ5B81.Z; ᵈ6A02.Z; ᵉBA00.Y, BA00.Z; ᶠLD40.0; ᵍ8D0Z; ʰ 5C3Z; ⁱ LB9Z; ʲ FB1Z; ᵏ6E8Z; ˡBC43.0, BC43.1, BC4Z; ᵐQC66; ⁿ5A14, 5A11; ᵒ5A00.Z

### Sex, degree of ID, HL, age

*Sex:* Trisomy 21, nutritional disorders, myocardial diseases, and hypothyroidism showed a female preponderance, while ASD exhibited a male preponderance. *Degree of ID:* Epilepsy, ASD, neuromuscular diseases, skeletal anomalies, spinal conditions, and myocardial diseases increased with higher degrees of ID. *HL:* HL was associated with visual impairment, trisomy 21, neuromuscular disorders, spinal conditions, and hypothyroidism, while ASD was inversely associated with HL. *Age:* Visual impairment, hypertension, obesity, mental, behavioural, or neurodevelopmental disorders, and diabetes increased with age. Hypothyroidism showed the highest prevalence in the middle-aged group. ASD, trisomy 21, and family history of eye or ear disorders exhibited a reversed age association.

## Discussion

We assessed the medical history of individuals with ID with and without manifest HL in order to evaluate (1) which comorbidities typically occur in people with ID and HL, (2) which of them occur in higher proportions in those with and without HL, and (3) whether severity of ID or HL influenced quantity of diseases, surgeries, or medication intake. To our knowledge, no existing literature compares disease occurrence in individuals with ID with and without HL. Our findings, which addressed age and sex, and systematised the data according to ICD-11, are a timely step toward better understanding this overlooked population within individuals with ID. This is especially important given that existing literature points to higher disease occurrence, morbidity, mortality, and undetected HL in individuals with ID in general. Examining and comparing these intersections adds knowledge about this group and their distinct needs.

Our findings suggest a higher prevalence of disease in individuals with both ID and HL than in those without HL for the majority of diseases assessed, thereby complementing and extending the existing literature reporting higher morbidity in individuals with ID [3–14] or HL [37]. Eight subcategories in the HL subcohort showed a higher prevalence with OR and 95% CI > 1: visual impairment; hypertension; trisomy 21; obesity; mental, behavioural, or neurodevelopmental disorders; spinal conditions; diabetes; and hypothyroidism. Among these eight, logistic regression highlighted the following: (1) associations between HL and visual impairment, trisomy 21, spinal conditions, hypothyroidism; (2) hypertension, obesity, and diabetes as being predominantly associated with increasing age; and (3) female sex correlated with trisomy 21 and hypothyroidism.

The overall high prevalence of visual impairment in our cohort stands in stark contrast to the prevalence of visual impairment reported for the adult general population in Germany (0.37%) [38]. Visual impairment was the most common comorbidity in both the overall cohort and the subcohort with ID and HL, aligning with previous research in individuals with ID [7]. This highlights the importance of multi-sensory impairment in individuals with ID. Sensory impairments, especially when co-occurring with other diseases, can be overlooked in individuals with ID – a phenomenon described as "diagnostic overshadowing" [39]. We detected visual system diseases in 22.8% of the HL subcohort, which supports the 21.4% prevalence of combined visual impairment and HL found in an Austrian study [39, 40]. Contrary to existing findings [41–43], our data did not replicate an association between visual impairment and higher degrees of ID, though it shows an increase with age [43]. Our observed association of the intersection of age, HL, and visual impairment mirrors the established evidence that sensory organs are more severely affected by aging than other types of organs [39]. The common co-occurrence of these sensory deficits likely also reflects shared pathophysiological pathways or frequent genetic aetiologies, rather than merely being a coincidental accumulation of age- or ID-related morbidity. A well-characterised example is Usher syndrome, in which mutations in genes (e.g. MYO7A) disrupt structures essential to both mechanotransduction in cochlear hair cells and function of photoreceptor/retinal pigment epithelium. As a result, individuals with Usher syndrome can exhibit combined vision impairment and HL [44].

Trisomy 21 occurred in 9.35% of our overall cohort, substantially higher compared to the general population estimates in Germany of 0.6% [45]. Trisomy 21, the most common known cause of ID [46], is an established risk factor for HL [23]. Our data showed a three- to fourfold higher occurrence of trisomy 21 in individuals with HL than in those without, and a female preponderance. Trisomy 21 showed associations with HL and high rates of visual diseases, as well as cardiovascular and metabolic co-occurring diseases (obesity, hypothyroidism, myocardial diseases, nutritional disorders, hypertension, dyslipidaemia). A 67% rate of manifest HL indicates pronounced (multi-)sensory impairment and aligns with existing findings on high rates of obesity, hypertension, and visual and hearing impairment in individuals with ID and trisomy 21 [7]. The high prevalence of HL in trisomy 21 has traditionally been attributed to craniofacial anatomical features predisposing to otitis media with effusion (OME), such as a small nasopharynx, narrow palate and (oro-)pharynx, stenotic external ear canal, dysplasia of the inner ear, flat nasal bridge, and malformation of the eustachian tube [47]. However, more recent work has also identified specific genetic drivers (e.g. DYRK1A) that promote middle-ear mucosal inflammation and effusion [48].

Hypothyroidism has been associated with HL and female sex in the general population [49]. We also observed this association in our analysis for the population with ID, suggesting links between hypothyroidism, HL, and female sex. The association between hypothyroidism and HL is pathophysiologically plausible, given the essential role of thyroid hormones in the development and function of the cochlea, including the differentiation and maturation of sensory hair cells (electromotility and potassium ion channel development), expression of ion channels, and synaptic maturation [50–52]. Thyroid hormone deficiency can directly impair these processes, for example by inducing β-tectorin protein deficiency, leading to structural abnormalities of the tectorial membrane and thereby predisposing to HL [52, 53].

Epilepsy occurred in similar proportions in both subcohorts (with HL 12.9%, without HL 12.3%) as one of the top 5 diseases, confirming its known relevance in individuals with ID [8], especially in contrast to prevalence estimates reported in the adult general population in Germany (0.95 to 1.09%) [54].

ASD was the most common disease subcategory in our cohort with ID and no HL (14.4%), with a clear male preponderance, aligning with previous observations [55, 56]. The occurrence of ASD within our cohort (10.4% overall, 14.4% without HL, 4.6% with HL) is notably higher than global estimates for the general population (0.65%) [57]. Studies on HL prevalence in ASD are scarce [39]; in our cohort, HL was less associated with ASD in both descriptive and regression analyses than in the sub-cohort without HL. Theories regarding the biological and sociological reasons underlying sex differences in ASD are subjects of ongoing discussion, and more research integrating critical approaches is necessary in order to illuminate this area [58].

A small association was observed between the number of comorbidities and greater severity of HL and ID, selectively in females, which supports existing evidence linking multimorbidity and female sex in both general [59] and ID populations [5]. This suggests an intersectional effect – overlapping female sex, ID, HL, and further diseases – and warrants further attention. Severity of HL also suggested an association between increasing degree of HL with medication intake in females.

Multimorbidity (more than two chronic diseases [36, 60]) was observed in nearly half of the cohort and was 21% higher in those with ID and HL (63%) than in without (42%). Existing evidence has found a similar rate of multimorbidity in individuals with ID (40% in Cooper et al. 2015 [5]), whereas multimorbidity in ID and HL has not been reported to date.

Multimorbidity and polypharmacy are strong predictors of mortality in older individuals with ID [61]. This underlines the need for early disease assessment and management in order to prevent progression and complications. Multimorbidity is complex, aetiologically influenced by age and sociocultural factors [60], and requires sustainable, cross-sectoral care that accounts for multifaceted lived realities.

40% of our cohort had manifest HL, 72.5% of which was undetected prior to the study. Dutch [23], German [62], and Austrian [40] studies reported high HL prevalence in individuals with ID (35.8%, 37.8%, 47%) and frequent lack of prior detection (47.6%, 28%, 72.8%). Despite differing scopes, the key implication of these studies is the need for systematic HL assessment in individuals with ID in order to detect and treat HL – and thereby other possible comorbidities – as early as possible.

### Limitations

Despite careful study design in cooperation with individuals with ID, caregivers, and their environment, a truly participatory approach with more active and equal roles would have added value. Medical history was collected orally and in writing, but would ideally have been complemented by further specific physical examinations and diagnostics [7], which were unfortunately not possible because of limited resources. Furthermore, the absence of a control group limits comparative analyses between clinical and outreach settings. The fact that the clinical control group did not participate ultimately underscores one of the core outcomes of our project overall: the critical necessity of outreach screening, as people with ID remain systematically underserved in traditional clinical settings [26, 63]. Transient HL was not included in the present analysis, but would warrant further investigation because of the risk of developing into manifest HL over time. Nevertheless, for our present analysis, the exclusion of participants with transient HL from the final analysis is unlikely to have strongly influenced the overall findings. Data from T1 showed that the prevalence of newly occurring HL at T1 (6.5%) was nearly balanced by the prevalence of HL that had resolved since T0 (7.0%). This suggests that the bidirectional fluctuations in hearing status largely offset each other. While this approach may potentially overlook individuals with highly recurrent conditions, such as repeated otitis media with effusion, the longitudinal screening design of the program is specifically intended to identify such patterns over time. Despite adjustment for age, sex, and severity of ID, residual confounding due to unmeasured factors such as care setting, socioeconomic status, and utilisation of healthcare cannot be ruled out. As all p-values were interpreted in an exploratory manner without adjustment for multiple testing, findings should be considered hypothesis-generating rather than confirmatory or causal, and should therefore be interpreted with caution. This cohort was recruited within a specific insurance context and geographic region (North Rhine-Westphalia), which may limit the generalisability of findings to broader populations, including those without statutory health insurance or in other healthcare systems. These limitations suggest avenues for potential future research and innovation.

The extent of co-occurring diseases, multimorbidity, and multiplied medication intake in participants with ID (and HL) emphasises the need for targeted attention and multidisciplinary, cross-sectoral healthcare. Each newly-diagnosed disease should serve as an indicator for further diagnostics in order to facilitate prevention and early intervention. General practitioners have the distinct responsibility, as long-term primary physicians, to integrate medical history and disease management with early referral to specialist care. This could be facilitated by the implementation of a cross-sectoral digital patient record within Germany in order to prevent information loss and ensure continuous, coordinated care. Increased awareness of the associations between ID, HL, and various distinct comorbidities should encourage general practitioners and specialists – especially paediatricians, ear, nose and throat (ENT) specialists, and specialists in phoniatrics and paediatric audiology – to pay particular attention to these interconnections. This would include, for example, considering screening for HL and other comorbidities for all individuals with ID. Clinical practice guidelines could serve as tools for fostering awareness and facilitating the comprehensive uptake of hearing screening within clinical care – especially for underserved populations [64].

Recent efforts to create and critically evaluate targeted diagnostics and care for diverse, underserved populations reflect a much-needed patient-centred approach [65–70]. For example, behavioural similarities between individuals with ASD, ID, and sensory impairment can complicate their differentiation at assessment. Diagnostic tools to aid this work have been proposed [68]. Sun et al. highlight the need for specific considerations regarding intervention as well as systematic seizure management in individuals with ID and epilepsy by developing a framework for targeted care which may serve as a good practice example [69]. One way to incentivise a multidisciplinary approach and foster structural change may involve resource allocation, e.g. monetary compensation.

Ableism (discrimination and devaluation on account of disabilities [71]) is societally-embedded at the structural, institutional, and individual level, including within healthcare. Individuals with ID and HL, often subsumed under the umbrella of so-called “disabilities”, face barriers and discrimination. As marginalised populations, they require heightened sensitivity, awareness, multidisciplinary care, and representative, disaggregated data that accurately capture their reality.

## Conclusion

Our findings identified (1) visual system diseases, epilepsy, and obesity as the most common comorbidities, with a high prevalence across the entire cohort. Concerning (2) the difference in prevalence of comorbidities between those with and without HL, our findings suggest increased comorbidity, multimorbidity, and higher intake of medication in individuals with HL than those without. Nearly all comorbidities observed in participants with HL (prevalence > 5% [= 14]) were more frequent in participants with HL vs. without (13/14), eight showing OR and 95% CI > 1: visual impairment; hypertension; trisomy 21; obesity; mental, behavioural, or neurodevelopmental disorders; spinal conditions; diabetes; hypothyroidism. Regarding (3) the influence of the degree of ID or HL, we found that higher degrees of ID are associated with an increased number of comorbidities, particularly epilepsy, ASD, neuromuscular, skeletal, spinal, and myocardial diseases. Beyond these associations, our results indicate that individuals with ID also seem to face compounded healthcare barriers – including data gaps, ableism, communication barriers, and limited access to care. Heightened awareness of the complex interplay between ID, HL, and their distinct comorbidities must translate into clinical practice. This entails general practitioners and specialists – particularly paediatricians, ENT-physicians, and specialists in phoniatrics and paediatric audiology – being especially attuned to these interconnections. Progress towards more equitable healthcare requires systematic implementation of differentiated, inclusive data generation, and distinct strategies in order to promote targeted, inclusive, multidisciplinary, person-centred, cross-sectoral care.

## Supplementary Information

Supplementary Material 1.

## Data Availability

The data underlying this study cannot be made publicly available because they contain sensitive personal information from a vulnerable population and were collected under informed consent provisions that restrict data sharing. The data are therefore subject to ethical and legal data protection requirements. The Competence Center for Clinical Trials Bremen (KKSB), University of Bremen, Germany (kks@math.uni-bremen.de), is the data-holding institution. Researchers wishing to validate the published findings may request access through the KKSB. A data dictionary and sample data will then be provided to enable researchers to develop their analysis code, which will be executed by KKSB staff on the original dataset. The resulting outputs will be returned to the requesting researchers. Access is limited to reproducing and validating the analyses reported in this publication; additional analyses are not permitted. KKSB staff are available for consultation if needed. The descriptive tables on which the results of this study rely are summarised and provided as supplementary material.

## Abbreviations

CI: Confidence interval
dB: Decibel
HL: Hearing loss
ICD-11: International Classification of Diseases, 11th Revision
ID: Intellectual disabilities
kHz: Kilohertz
OR: Odds ratio
WHO: World Health Organization
Yrs: Years
