# Supplementary material for: Comorbidities in individuals with intellectual disabilities with and without hearing loss: a prospective cohort study
Source: BMC Public Health. 2026 Sep 26;26:3306. doi: 10.1186/s12889-026-29321-w (PMC13749877; doi:10.1186/s12889-026-29321-w)
Supplement: Supplementary file 1 [file 12889_2026_29321_MOESM1_ESM.pdf]

Comorbidities of individuals with intellectual disabilities with and without hearing loss: a prospective cohort study – Supplementary material

Table A.1. Age and hearing status in the study population.

**Age group**	**0-5** N = 231	**6-11** N = 249	**12-17** N = 156	**18-34** N = 108	**35-49** N = 92	**50-69** N = 181	**>=70** N = 36	**Overall** N = 1,053
Age								
Mean	4	8	14	24	42	59	75	24
SD	1	2	2	5	5	5	5	22
Median (Q1, Q3)	4 (3, 5)	8 (7, 10)	14 (13, 16)	22 (19, 28)	42 (38, 46)	59 (54, 63)	73 (72, 77)	13 (6, 42)
Min, Max	1, 5	6, 11	12, 17	18, 34	35, 49	50, 69	70, 90	1, 90
Hearing loss (manifest)								
yes	52 (23%)	64 (26%)	38 (24%)	28 (26%)	57 (62%)	144 (80%)	34 (94%)	417 (40%)
no	147 (64%)	159 (64%)	110 (71%)	73 (68%)	30 (33%)	34 (19%)	1 (2.8%)	554 (53%)
unclear	32 (14%)	26 (10%)	8 (5.1%)	7 (6.5%)	5 (5.4%)	3 (1.7%)	1 (2.8%)	82 (7.8%)
Hearing status (differentiated)								
HL (manifest)	52 (23%)	64 (26%)	38 (24%)	28 (26%)	57 (62%)	144 (80%)	34 (94%)	417 (40%)
HL (transient)	31 (13%)	30 (12%)	13 (8.3%)	18 (17%)	7 (7.6%)	11 (6.1%)	1 (2.8%)	111 (11%)
No HL	116 (50%)	129 (52%)	97 (62%)	55 (51%)	23 (25%)	23 (13%)	0 (0%)	443 (42%)
HL (unclear whether manifest or transient)	3 (1.3%)	3 (1.2%)	3 (1.9%)	0 (0%)	0 (0%)	0 (0%)	0 (0%)	9 (0.9%)
Hearing status not sufficiently clarified	29 (13%)	23 (9.2%)	5 (3.2%)	7 (6.5%)	5 (5.4%)	3 (1.7%)	1 (2.8%)	73 (6.9%)

Table A.2. Occurences of disease categories stratified by sex.

Disease category	Male N=662	Female N=390	Total N=1053
05 Endocrine, nutritional or metabolic diseases	151 (22.8%)	117 (30%)	268 (25.5%)
20 Developmental anomalies	159 (24%)	104 (26.7%)	263 (25%)
08 Diseases of the nervous system	155 (23.4%)	89 (22.8%)	244 (23.2%)
06 Mental, behavioural or neurodevelopmental disorders	160 (24.2%)	83 (21.3%)	243 (23.1%)
09 Diseases of the visual system	107 (16.2%)	80 (20.5%)	187 (17.8%)
11 Diseases of the circulatory system	93 (14%)	79 (20.3%)	172 (16.3%)
15 Diseases of the musculoskeletal system or connective tissue	51 (7.7%)	48 (12.3%)	99 (9.4%)
21 Symptoms, signs or clinical findings, not elsewhere classified	52 (7.9%)	32 (8.2%)	84 (8%)
10 Diseases of the ear or mastoid process	32 (4.8%)	25 (6.4%)	57 (5.4%)
24 Factors influencing health status or contact with health services	40 (6%)	17 (4.4%)	57 (5.4%)
12 Diseases of the respiratory system	26 (3.9%)	12 (3.1%)	38 (3.6%)
13 Diseases of the digestive system	10 (1.5%)	10 (2.6%)	20 (1.9%)
04 Diseases of the immune system	7 (1.1%)	6 (1.5%)	13 (1.2%)
14 Diseases of the skin	6 (0.9%)	4 (1%)	10 (0.9%)
19 Certain conditions originating in the perinatal period	6 (0.9%)	2 (0.5%)	8 (0.8%)
03 Diseases of the blood or blood-forming organs	2 (0.3%)	3 (0.8%)	5 (0.5%)
07 Sleep-wake disorders	4 (0.6%)	1 (0.3%)	5 (0.5%)
16 Diseases of the genitourinary system	4 (0.6%)	1 (0.3%)	5 (0.5%)
01 Certain infectious or parasitic diseases	4 (0.6%)	0 (0%)	4 (0.4%)
02 Neoplasms	2 (0.3%)	2 (0.5%)	4 (0.4%)
22 Injury, poisoning or certain other consequences of external causes	1 (0.2%)	1 (0.3%)	2 (0.2%)

Table A.3. Occurences of the most common diseases in the overall cohort stratified by sex.

Disease subcategory	Male N=662	Female N=390	Total N=1053
Diseases of the visual system and vision impairment	106 (16%)	80 (20.5%)	186 (17.7%)
Epilepsy or seizures, unspecified	83 (12.5%)	47 (12.1%)	130 (12.3%)
Obesity	64 (9.7%)	60 (15.4%)	124 (11.8%)
Autism spectrum disorder (ASD)	87 (13.1%)	23 (5.9%)	110 (10.4%)
Hypertension	57 (8.6%)	46 (11.8%)	103 (9.8%)
Complete trisomy 21	50 (7.6%)	48 (12.3%)	98 (9.3%)
Diseases of neuromuscular junction or muscle	59 (8.9%)	37 (9.5%)	96 (9.1%)
Nutritional disorders	51 (7.7%)	42 (10.8%)	93 (8.8%)
Structural developmental anomalies of the skeleton	52 (7.9%)	41 (10.5%)	93 (8.8%)
Conditions associated with the spine	38 (5.7%)	32 (8.2%)	70 (6.6%)
Mental, behavioural or neurodevelopmental disorders, unspecified	31 (4.7%)	33 (8.5%)	64 (6.1%)
Diseases of the myocardium or cardiac chambers and cardiomyopathy	30 (4.5%)	33 (8.5%)	63 (6%)
Family history of eye or ear disorders	38 (5.7%)	17 (4.4%)	55 (5.2%)
Diabetes	24 (3.6%)	21 (5.4%)	45 (4.3%)
Hypothyroidism	16 (2.4%)	24 (6.2%)	40 (3.8%)

Table A.4. Occurences of the most common diseases in the cohort with HL stratified by sex.

Disease subcategory	Male N=248	Female N=169	Total N=417
Diseases of the visual system and vision impairment	52 (21%)	43 (25.4%)	95 (22.8%)
Hypertension	43 (17.3%)	32 (18.9%)	75 (18%)
Obesity	35 (14.1%)	36 (21.3%)	71 (17%)
Complete trisomy 21	31 (12.5%)	35 (20.7%)	66 (15.8%)
Epilepsy or seizures, unspecified	37 (14.9%)	17 (10.1%)	54 (12.9%)
Diseases of neuromuscular junction or muscle	29 (11.7%)	17 (10.1%)	46 (11%)
Structural developmental anomalies of the skeleton	27 (10.9%)	18 (10.7%)	45 (10.8%)
Mental, behavioural or neurodevelopmental disorders	20 (8.1%)	22 (13%)	42 (10.1%)
Conditions associated with the spine	26 (10.5%)	15 (8.9%)	41 (9.8%)
Nutritional disorders	22 (8.9%)	16 (9.5%)	38 (9.1%)

Disease subcategory	**Male**<br>N=248	**Female**<br>N=169	**Total**<br>N=417
Diabetes	20 (8.1%)	15 (8.9%)	35 (8.4%)
Diseases of the myocardium or cardiac chambers and cardiomyopathy	17 (6.9%)	14 (8.3%)	31 (7.4%)
Hypothyroidism	14 (5.6%)	16 (9.5%)	30 (7.2%)
Family history of eye or ear disorders	13 (5.2%)	10 (5.9%)	23 (5.5%)
Autism spectrum disorder (ASD)	15 (6%)	4 (2.4%)	19 (4.6%)

Table A.5. Occurences of the most common diseases in the cohort without HL stratified by sex.

**Disease subcategory**	**Male**<br>N=353	**Female**<br>N=200	**Total**<br>N=554
Autism spectrum disorder (ASD)	62 (17.6%)	18 (9%)	80 (14.4%)
Diseases of the visual system and vision impairment	41 (11.6%)	35 (17.5%)	76 (13.7%)
Epilepsy or seizures, unspecified	42 (11.9%)	26 (13%)	68 (12.3%)
Obesity	29 (8.2%)	24 (12%)	53 (9.6%)
Nutritional disorders	23 (6.5%)	24 (12%)	47 (8.5%)
Diseases of neuromuscular junction or muscle	26 (7.4%)	17 (8.5%)	43 (7.8%)
Structural developmental anomalies of the skeleton	20 (5.7%)	20 (10%)	40 (7.2%)
Family history of eye or ear disorders	24 (6.8%)	7 (3.5%)	31 (5.6%)
Diseases of the myocardium or cardiac chambers and cardiomyopathy	12 (3.4%)	18 (9%)	30 (5.4%)
Hypertension	14 (4%)	13 (6.5%)	27 (4.9%)
Complete trisomy 21	13 (3.7%)	13 (6.5%)	26 (4.7%)
Conditions associated with the spine	11 (3.1%)	14 (7%)	25 (4.5%)
Mental, behavioural or neurodevelopmental disorders	8 (2.3%)	8 (4%)	16 (2.9%)
…			
Hypothyroidism	2 (0.6%)	8 (4%)	10 (1.8%)
…			
Diabetes	3 (0.8%)	5 (2.5%)	8 (1.4%)

Table A.6. Odds ratios regarding HL and most common diseases in the overall cohort.

Disease subcategory	**Total** N=971		
	**HL** N_1=417	**No HL** N_2=554	**OR (95%-CI)**
Diseases of the visual system and vision impairment	95	76	1.86 (1.3,2.6)
Obesity	71	53	1.94 (1.3,2.9)
Epilepsy or seizures, unspecified	54	68	1.06 (0.7,1.6)
Hypertension	75	27	4.28 (2.7,6.8)
Autism spectrum disorder (ASD)	19	80	0.28 (0.1,0.5)
Complete trisomy 21	66	26	3.82 (2.3,6.2)
Diseases of neuromuscular junction or muscle	46	43	1.47 (0.9,2.3)
Nutritional disorders	38	47	1.08 (0.6,1.7)
Structural developmental anomalies of the skeleton	45	40	1.55 (0.9,2.5)
Conditions associated with the spine	41	25	2.31 (1.3,3.9)
Diseases of the myocardium or cardiac chambers and cardiomyopathy	31	30	1.4 (0.8,2.4)
Mental, behavioural or neurodevelopmental disorders	42	16	3.77 (2,6.8)
Family history of eye or ear disorders	23	31	0.98 (0.5,1.8)
Diabetes	35	8	6.25 (2.8,13.6)
Hypothyroidism	30	10	4.22 (2,8.8)

Table A.7. Overview of the subcohort with Trisomy 21.

**Variables**	**N = 98**
Sex	
male	50 (51%)
female	48 (49%)
Age	

Variables	N = 98
Mean	25
SD	22
Median (Q1, Q3)	14 (6, 48)
Min, Max	2, 78
Hearing loss (manifest)	
yes	66 (67%)
no	26 (27%)
unclear	6 (6.1%)
Conductive hearing loss	
yes	24 (24%)
no	65 (66%)
unclear	5 (5.1%)
(missing)	4 (4.1%)
Sensorineural hearing loss	
yes	18 (18%)
no	70 (71%)
unclear	6 (6.1%)
(missing)	4 (4.1%)
Mixed hearing loss	
yes	10 (10%)
no	78 (80%)
unclear	6 (6.1%)
(missing)	4 (4.1%)
Unspecified hearing loss	
yes	18 (18%)
no	70 (71%)
unclear	6 (6.1%)
(missing)	4 (4.1%)

Table A.8. Most common diseases in the subcohort of people with trisomy 21.

Disease subcategory	Frequency	Percent
Complete trisomy 21	98	100.0
Diseases of the visual system and vision impairment	23	23.5
Obesity	21	21.4
Hypothyroidism	12	12.2
Diseases of the myocardium or cardiac chambers and cardiomyopathy	12	12.2
Nutritional disorders	8	8.2
Diseases of neuromuscular junction or muscle	7	7.1
Hypertension	7	7.1
Diseases of middle ear or mastoid	6	6.1
Structural developmental anomalies of the skeleton	6	6.1
Unspecified disorders of lipoprotein metabolism or lipidaemias	5	5.1
Conditions associated with the spine	5	5.1

Table A.9. Number of comorbidities, medications and surgeries stratified by HL.

Variables	With HL N = 417	Without HL N = 554	Overall N = 1,053
Number of comorbidities[1]			
Mean	2.75	1.65	2.07
SD	2.35	1.87	2.17
Median (Q1, Q3)	2.00 (1.00, 4.00)	1.00 (0.00, 3.00)	1.00 (0.00, 3.00)
Min, Max	0.00, 14.00	0.00, 10.00	0.00, 14.00
(missing)	0 (0%)	0 (0%)	0 (0%)
Number of comorbidities (categories)[2]			
0	67 (16%)	194 (35%)	293 (28%)
1	87 (21%)	128 (23%)	236 (22%)
2+	263 (63%)	232 (42%)	524 (50%)
Number of medications[3]			
Mean	2.73	0.84	1.62
SD	3.04	1.56	2.46
Median (Q1, Q3)	2.00 (0.00, 4.00)	0.00 (0.00, 1.00)	0.00 (0.00, 2.00)

Variables	With HL<br>N = 417	Without HL<br>N = 554	Overall<br>N = 1,053
Min, Max	0.00, 15.00	0.00, 12.00	0.00, 15.00
(missing)	36 (8.6%)	51 (9.2%)	98 (9.3%)
Number of surgeries[4]			
Mean	1.00	0.65	0.80
SD	1.30	1.06	1.19
Median (Q1, Q3)	1.00 (0.00, 2.00)	0.00 (0.00, 1.00)	0.00 (0.00, 1.00)
Min, Max	0.00, 7.00	0.00, 8.00	0.00, 8.00
(missing)	38 (9.1%)	50 (9.0%)	99 (9.4%)

[1] Welch test: t = 7.872, df = 774.5, p-value < 0.001
[2] Chi-squared test for binary variable (2+ vs. 0/1): Chi-squared = 41.91, df = 1, p-value < 0.001
[3] Welch test: t = 11.08, df = 531.2, p-value < 0.001
[4] Welch test: t = 4.227, df = 714.8, p-value < 0.001

Table A.10. Correlation regarding degree of ID and number of comorbidities, medication intake and number of surgeries within people with HL.

Subgroup	N[1]	Spearman's Rho (95%-CI)
**Number of comorbidities**		
Overall	388	0.069 (-0.031,0.167)
Male	230	0.012 (-0.117,0.141)
Female	158	0.175 (0.019,0.322)
**Medication intake**		
Overall	388	0.034 (-0.07,0.137)
Male	230	0.011 (-0.124,0.147)
Female	158	0.077 (-0.086,0.236)
**Number of surgeries**		
Overall	388	-0.026 (-0.129,0.079)
Male	230	-0.02 (-0.155,0.117)
Female	158	-0.04 (-0.202,0.123)

[1] Number of people with HL and no missing entry for degree of ID in the respective subgroup

Table A.11. Correlation regarding grade of HL and number of comorbidities, medication intake and number of surgeries within people with HL.

Subgroup	N[1]	Spearman's Rho (95%-CI)
**Number of comorbidities**		
Overall	392	0.086 (-0.013,0.183)
Male	237	0.025 (-0.102,0.152)
Female	155	0.192 (0.036,0.34)
**Medication intake**		
Overall	392	0.162 (0.06,0.262)
Male	237	0.134 (0,0.263)
Female	155	0.207 (0.044,0.359)
**Number of surgeries**		
Overall	392	0.051 (-0.053,0.153)
Male	237	0.068 (-0.067,0.2)
Female	155	0.024 (-0.141,0.188)

[1] Number of people with HL and no missing entry for grade of HL in the respective subgroup

Table A.12. Correlation regarding degree of ID and number of comorbidities, medication intake and number of surgeries within people without HL.

Subgroup	N[1]	Spearman's Rho (95%-CI)
**Number of comorbidities**		
Overall	522	0.166 (0.082,0.249)
Male	335	0.156 (0.049,0.259)
Female	186	0.182 (0.039,0.317)
**Medication intake**		
Overall	522	0.118 (0.029,0.206)
Male	335	0.149 (0.038,0.257)
Female	186	0.061 (-0.09,0.208)
**Number of surgeries**		
Overall	522	0.139 (0.05,0.226)
Male	335	0.187 (0.077,0.293)
Female	186	0.069 (-0.082,0.217)

[1] Number of people without HL and no missing entry for degree of ID in the respective subgroup

Table A.13. Cross table of previously known HL and detected HL.

	Manifest HL			
	Yes	No	Unclear	**Total**
**HL previously known**				
Yes	110 (11%)	30 (3.0%)	7 (0.7%)	147 (15%)
No	290 (29%)	498 (50%)	68 (6.8%)	856 (85%)
**Total**	400 (40%)	528 (53%)	75 (7.5%)	1,003 (100%)

Table A.14. Previously overlooked HL by sex.

**Sex**	**Male** N = 662	**Female** N = 390
**HL previously overlooked**		
Yes	174 (26%)	116 (30%)
No	402 (61%)	235 (60%)
Unclear	55 (8.3%)	20 (5.1%)
(missing)	31 (4.7%)	19 (4.9%)

Chi-squared test (unclear and missing values excluded): Chi-squared = 0.692, df = 1, p-value = 0.406

Colour-adjusted figure legends for improved accessibility (modified for individuals with red-green colour vision deficiency)

Figure 1. All 21 ICD-11 disease categories in the overall population, contrasting HL and sex differences. Percentual sex differences are highlighted within each disease category. The white vertical line marks the position, in which sex distribution among individuals with the disease would match that of the overall cohort or the respective subcohort (with/without HL), representing the expected ratio if the diseases were independent of sex. If the white vertical line falls within either the purple or blue area, it indicates a higher disease prevalence in that sex.

Figure 2. **Comparison of top 14 most common disease subcategories in participants with and without HL.** Upper row: order in participants without HL. Lower row: order in participants with HL. Colour frames indicate each disease's position per group. Straight lines show position changes, dotted lines indicate diseases exiting the top 14 list. Background boxes show sex differences: blue = female,

purple = male. Eight differening conditions showing OR and 95% CI >1 between both groups are highlighted in HL row in separate boxes.

Figure 3. Number of diseases in participants with and without HL.

Figure 1.

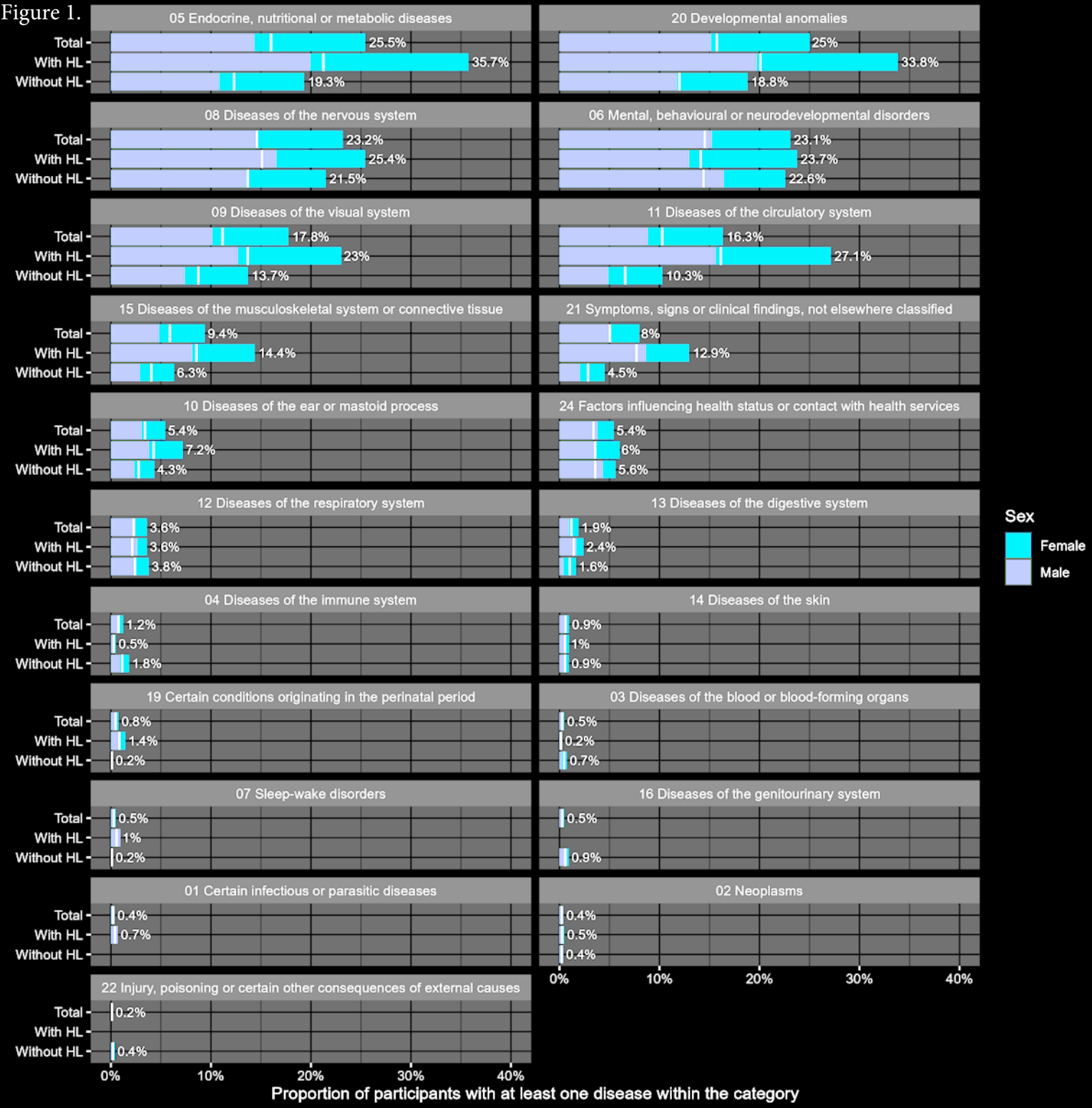

05 Endocrine, nutritional or metabolic diseases
Total 25.5%
With HL 35.7%
Without HL 19.3%
20 Developmental anomalies
Total 25%
With HL 33.8%
Without HL 18.8%
08 Diseases of the nervous system
Total 23.2%
With HL 25.4%
Without HL 21.5%
06 Mental, behavioural or neurodevelopmental disorders
Total 23.1%
With HL 23.7%
Without HL 22.6%
09 Diseases of the visual system
Total 17.8%
With HL 23%
Without HL 13.7%
11 Diseases of the circulatory system
Total 16.3%
With HL 27.1%
Without HL 10.3%
15 Diseases of the musculoskeletal system or connective tissue
Total 9.4%
With HL 14.4%
Without HL 6.3%
21 Symptoms, signs or clinical findings, not elsewhere classified
Total 8%
With HL 12.9%
Without HL 4.5%
10 Diseases of the ear or mastoid process
Total 5.4%
With HL 7.2%
Without HL 4.3%
24 Factors influencing health status or contact with health services
Total 5.4%
With HL 6%
Without HL 5.6%
12 Diseases of the respiratory system
Total 3.6%
With HL 3.6%
Without HL 3.8%
13 Diseases of the digestive system
Total 1.9%
With HL 2.4%
Without HL 1.6%
04 Diseases of the immune system
Total 1.2%
With HL 0.5%
Without HL 1.8%
14 Diseases of the skin
Total 0.9%
With HL 1%
Without HL 0.9%
19 Certain conditions originating in the perinatal period
Total 0.8%
With HL 1.4%
Without HL 0.2%
03 Diseases of the blood or blood-forming organs
Total 0.5%
With HL 0.2%
Without HL 0.7%
07 Sleep-wake disorders
Total 0.5%
With HL 1%
Without HL 0.2%
16 Diseases of the genitourinary system
Total 0.5%
Without HL 0.9%
01 Certain infectious or parasitic diseases
Total 0.4%
With HL 0.7%
Without HL
02 Neoplasms
Total 0.4%
With HL 0.5%
Without HL 0.4%
22 Injury, poisoning or certain other consequences of external causes
Total 0.2%
With HL
Without HL 0.4%
0% 10% 20% 30% 40%
Proportion of participants with at least one disease within the category
Sex
Female
Male

Figure 2.

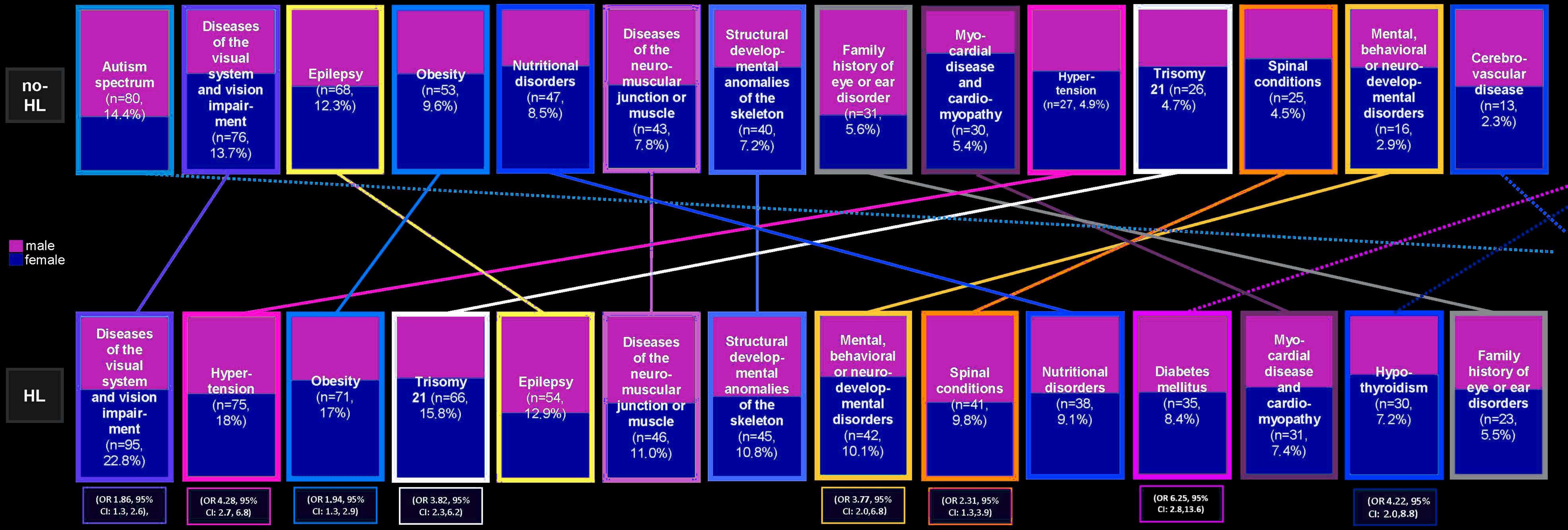
no-HL
HL
male
female
Autism spectrum (n=80, 14.4%)
Diseases of the visual system and vision impairment (n=76, 13.7%)
Epilepsy (n=68, 12.3%)
Obesity (n=53, 9.6%)
Nutritional disorders (n=47, 8.5%)
Diseases of the neuromuscular junction or muscle (n=43, 7.8%)
Structural developmental anomalies of the skeleton (n=40, 7.2%)
Family history of eye or ear disorder (n=31, 5.6%)
Myocardial disease and cardiomyopathy (n=30, 5.4%)
Hypertension (n=27, 4.9%)
Trisomy 21 (n=26, 4.7%)
Spinal conditions (n=25, 4.5%)
Mental, behavioral or neurodevelopmental disorders (n=16, 2.9%)
Cerebrovascular disease (n=13, 2.3%)
Diseases of the visual system and vision impairment (n=95, 22.8%)
Hypertension (n=75, 18%)
Obesity (n=71, 17%)
Trisomy 21 (n=66, 15.8%)
Epilepsy (n=54, 12.9%)
Diseases of the neuromuscular junction or muscle (n=46, 11.0%)
Structural developmental anomalies of the skeleton (n=45, 10.8%)
Mental, behavioral or neurodevelopmental disorders (n=42, 10.1%)
Spinal conditions (n=41, 9.8%)
Nutritional disorders (n=38, 9.1%)
Diabetes mellitus (n=35, 8.4%)
Myocardial disease and cardiomyopathy (n=31, 7.4%)
Hypothyroidism (n=30, 7.2%)
Family history of eye or ear disorders (n=23, 5.5%)
(OR 1.86, 95% CI: 1.3, 2.6),
(OR 4.28, 95% CI: 2.7, 6.8)
(OR 1.94, 95% CI: 1.3, 2.9)
(OR 3.82, 95% CI: 2.3,6.2)
(OR 3.77, 95% CI: 2.0,6.8)
(OR 2.31, 95% CI: 1.3,3.9)
(OR 6.25, 95% CI: 2.8,13.6)
(OR 4.22, 95% CI: 2.0,8.8)

Figure 3.

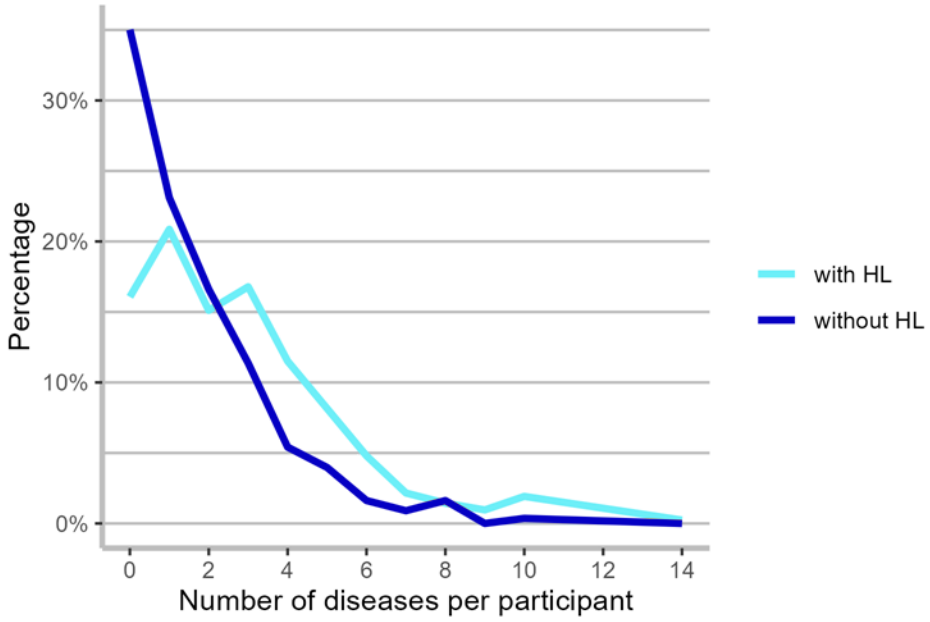

30%
20%
10%
0%
Percentage
0
2
4
6
8
10
12
14
Number of diseases per participant
with HL
without HL

Algorithm – Manifest Hearing Loss

Algorithm to determine the subgroup of individuals with manifest hearing loss (HL)

A manifest hearing loss is present if:

1. … a hearing loss is present at both t0 and t1.
2. … a hearing loss is present at t0 and hearing status at t1 is unclear (or a drop-out occurs).
3. … hearing is normal at t0 and a sensorineural, mixed, or unspecified hearing loss is present at t1.

In the latter case, tympanometry results must additionally indicate "no seal" or "normal peak," or no tympanometry must have been performed.

4. … hearing status is unclear at t0 and a sensorineural, mixed, or unspecified hearing loss is present at t1. In the latter case, tympanometry results must additionally indicate "no seal" or "normal peak," or no tympanometry must have been performed.

A manifest hearing loss is not present if:

1. … hearing is normal at t1.
2. … hearing is normal at t0 and either a pure conductive hearing loss or an unspecified hearing loss is present at t1. In the second case, tympanometry results must additionally indicate "flat" or "negative pressure."

Hearing status regarding a manifest hearing loss is unclear if:

1. … hearing status is unclear (or a drop-out occurs) at both t0 and t1.
2. … hearing is normal at t0 and hearing status is unclear (or a drop-out occurs) at t1.
3. … hearing status is unclear at t0 and either a pure conductive hearing loss or an unspecified hearing loss is present at t1, with tympanometry results additionally indicating "flat" or "negative pressure."
